# YOLOv4-Tiny-Based Coal Gangue Image Recognition and FPGA Implementation

**DOI:** 10.3390/mi13111983

**Published:** 2022-11-16

**Authors:** Shanyong Xu, Yujie Zhou, Yourui Huang, Tao Han

**Affiliations:** 1 School of Electrical & Information Engineering, Anhui University of Science and Technology, Huainan 232001, China; 2 School of Electrical and Opto Electronic Engineering, West Anhui University, Lu’an 237012, China

**Keywords:** coal gangue recognition, deep learning, FPGA, convolution, pooling, IP kernel designing

## Abstract

Nowadays, most of the deep learning coal gangue identification methods need to be performed on high-performance CPU or GPU hardware devices, which are inconvenient to use in complex underground coal mine environments due to their high power consumption, huge size, and significant heat generation. Aiming to resolve these problems, this paper proposes a coal gangue identification method based on YOLOv4-tiny and deploys it on the low-power hardware platform FPGA. First, the YOLOv4-tiny model is well trained on the computer platform, and the computation of the model is reduced through the 16-bit fixed-point quantization and the integration of a BN layer and convolution layer. Second, convolution and pooling IP kernels are designed on the FPGA platform to accelerate the computation of convolution and pooling, in which three optimization methods, including input and output channel parallelism, pipeline, and ping-pong operation, are used. Finally, the FPGA hardware system design of the whole algorithm is completed. The experimental results of the self-made coal gangue data set indicate that the precision of the algorithm proposed in this paper for coal gangue recognition on the FPGA platform are slightly lower than those of CPU and GPU, and the mAP value is 96.56%; the recognition speed of each image is 0.376 s, which is between those of CPU and GPU; the hardware power consumption of the FPGA platform is only 2.86 W; and the energy efficiency ratio is 10.42 and 3.47 times that of CPU and GPU, respectively.

## 1. Introduction

Gangue is a kind of main solid waste produced in the coal mining process. If it is not treated before coal combustion, the calorific value of coal is reduced, and the emission of harmful substances increases. Therefore, it is very important to identify and sort coal gangue cleanly and efficiently. Coal gangue sorting methods that are commonly used in the early stage include the ray method [1,2,3], impact crush method [4,5,6], laser method [7], and infrared thermal wave detection [8], etc., most of which are complicated in principle, expensive, and risky. Coal gangue recognition methods that are based on image processing are relatively novel with simple recognition steps, relatively cheap equipment, and high security; hence, they are currently the mainstream methods for coal gangue recognition. Such methods can mainly be divided into traditional image analysis methods and deep learning image processing methods.

Sun [9] adopted the morphological principle to identify the areas of interest on the surface of coal and gangue to extract texture and trace features and establish a coal and gangue classifier. Hobson [10] distinguished coal gangue by the texture features of images and analyzed the coal gangue texture using the gray-level co-occurrence matrix (GLCM). Ma [11] proposed a coal gangue image recognition method based on wavelet transform (WT), which adopted the embedded zero tree wavelets encoding (EZWE) algorithm to transmit the bit stream of coal gangue images, and the wavelet moment to extract the features of coal gangue images. Song [12] designed an online automatic coal gangue sorting system based on an improved BP algorithm and ARM by utilizing the BP neural network’s advantages of parallel computation, nonlinear mapping, and self-adaptation, and the ARM microcontroller’s characteristics of high performance, small volume, low power consumption, and low cost. Compared with traditional image analysis algorithms, the deep learning algorithm can automatically learn the features of coal gangue images, with higher recognition accuracy and recognition speed. Li [13] designed a CG-RPN network to determine the target areas of coal and gangue, and then constructed a convolutional neural network for coal and gangue identification. Zhang [14] improved the one-stage object detection algorithm SSD by replacing its backbone network with MobileNet to identify coal gangue images with the improved SSD algorithm. Alfarzaeai [15] designed a new convolutional neural network model to recognize coal gangue thermal images. Eshaq [16] also detected coal gangue infrared thermal images, and found that the detection results of the ResNet-18 and DenseNet-201 algorithms were the best after comparing the detection performance of multiple common deep learning algorithms. Pan. H [17] added spatial pyramid pooling, the attention mechanism module, and dilated convolution to the YOLOv3-tiny algorithm to identify coal gangue, which speeded up the identification while ensuring the accuracy. Gui [18] applied the more advanced YOLOv5 target detection algorithm and added an attention mechanism module to the original algorithm, which improved the coal gangue recognition accuracy. Deep learning features a large number of network layers and parameters and complex models. Currently, most of the deep learning acceleration tools are high-performance CPU or GPU devices with high power consumption, a large volume, and significant heat generation. This it difficult for them to be utilized in the complex underground environment of coal mines. However, as a new tool for deep learning algorithm implementation and acceleration, FPGA has many advantages, including a strong parallel computing capability, low power consumption, and small size [19,20,21,22]. Li [23] deployed the improved YOLO network algorithm on ZYNQ and used two optimization methods: fixed-point quantization and Relu function instead of the leaky function, which reduced the amount of network computing and saved FPGA chip resources. Wei [24] proposed a YOLO hardware accelerator based on the ARM + FPGA architecture. ARM and FPGA exchange data through the AXI bus. Yu [25] proposed an FPGA architecture consisting of three pipelining stages, each corresponding to a layer of the YOLOv3-tiny network. Li [26] used 16-bit fixed-point quantization and network pruning methods in the process of mapping YOLOv4-tiny to FPGA, which reduced the computational complexity and prevented the network from overfitting.

Coal gangue images mainly contain black and gray tones, and the texture features are relatively simple and only contain two types of targets. Therefore, the use of lightweight YOLO series algorithms can not only meet the requirements of the recognition accuracy but also achieve a faster recognition speed. This paper presents a YOLOv4-tiny-based coal gangue image recognition method and deploys it on a low-power hardware platform FPGA. ZYNQ-7020 is used for the FPGA platform, which is a heterogeneous platform with two parts: ARM and FPGA. The FPGA platform mainly includes convolution and pooling IP kernels, data input and output circuits for auxiliary computing, and on-chip cache to complete convolution, pooling, and other computationally intensive tasks. The ARM side mainly performs tasks with a relatively small amount of computation such as initialization of each module and image preprocessing, and completes the forward reasoning of the entire algorithm network by multiplexing convolution and pooling IP kernels. Because the YOLOv4-tiny algorithm involves a large number of convolution and pooling computations, the design of convolution and pooling IP kernels is critical in this study. To speed up the computation, this paper uses several optimization methods during the hardware deployment. In coal gangue image recognition, compared with GPU and CPU, the FPGA platform in this paper obtains the highest energy efficiency ratio.

## 2. Principle of the YOLOv4-Tiny Algorithm

The YOLO-tiny algorithm is lightweight as it realizes the lightweight of the network by compressing the network model structure, simplifying feature extraction, and integrating the algorithm steps. This process may cause a certain loss in the algorithm detection accuracy but can greatly improve the detection speed. Therefore, the YOLO-tiny algorithm is very suitable for deployment on embedded devices with insufficient computing power.

**Figure 1:**
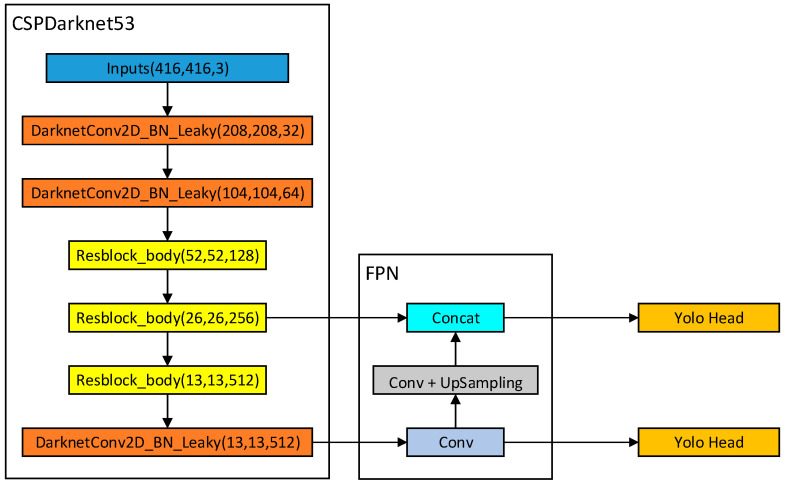
YOLOv4-tiny model structure.

After the YOLOv4 algorithm [27] was proposed in 2020, its lightweight version YOLOv4-tiny [28] was successively proposed. Compared with YOLOv4, YOLOv4-tiny has a lot of lightweight strategies in the backbone feature extraction network and FPN part and removes most structures of the algorithm in the deep network, resulting in a parameter amount of only one-tenth of that of YOLOv4. Figure 1 demonstrates the structure of the YOLOv4-tiny algorithm, in which the CSPdarknet53 is used as the backbone network for feature extraction, including only three convolutional layers with a convolution kernel size of 3 × 3, and a three-layer Resblock_body structure; the FPN part is a bottom-up feature pyramid structure that uses a single-layer convolution structure to upsample features. This algorithm only uses the last two Yolo Heads for feature regression to obtain classification results. Different Yolo Heads can detect targets of different sizes. The target size of coal gangue is relatively close, so the YOLOv4 tiny algorithm with two Yolo Heads is suitable for identifying coal gangue.

**Figure 2:**
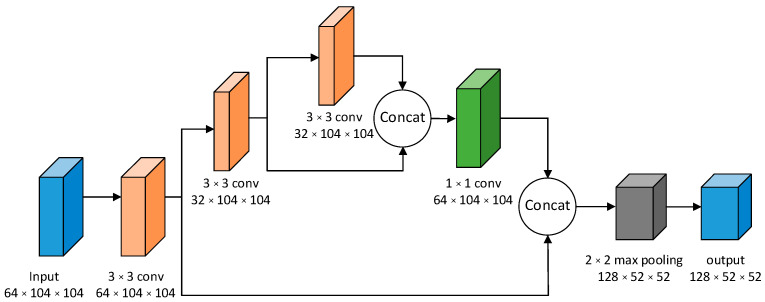
Resblock_body network structure.

DarknetConv2D_BN_Leaky consists of a 3 × 3 convolution, a batch normalization, and a leaky rule activation function. Figure 2 shows the structure of Resblock_body. Taking the first Resblock_body as an example, the input feature map size is 104 × 104 and the number of channels is 64. First, a 3 × 3 standard convolution is used to extract features. Then, the feature map is divided into two parts for feature extraction: the first part, Part A, takes half of the original 64 channels, and the second part takes all the original channels. A 3 × 3 convolution is performed in Part A and the channels are divided into two lines for operation. One line is performed with the 3 × 3 convolution again while the other line is not performed. Afterwards, channel stacking is carried out and a 1 × 1 convolution is performed for feature extraction. Later, all the results of the channels of both parts are stacked and, finally, a 2 × 2 maximum pooling layer with a step size of 2 is adopted to reduce the size of the feature map. The final feature map is output to the next stage, with a size of 52 × 52 and a number of channels of 128.

## 3. Optimization Method for Algorithm FPGA Implementation

When deploying complex neural network models to embedded platforms, it is necessary to use some optimization methods because the hardware resources of the embedded platforms are often limited. First, the computational load of the model is preliminarily reduced by integrating the computation of the BN layer and convolutional layer on the trained algorithm model. Second, the 16-bit fixed-point data are considered for the quantification of the model weight because the 32-bit floating-point weight parameters of the model trained on the computer platform can use many FPGA resources. Finally, as the computation in the YOLOv4-tiny algorithm is mainly for convolution and pooling, this paper designs convolution and pooling IP kernels to accelerate the algorithm computation. In the IP kernel design, three optimization methods including pipeline operation optimization, ping-pong operation, and parallel computation of input and output channels are applied to improve FPGA resource utilization and the computing efficiency. Detailed descriptions of these optimization methods are provided in the following sections.

### 3.1. Integration of the BN Layer and Convolution Layer

BN (batch normalization) is mainly used to solve the problems of training difficulty and slow convergence of the deep convolution neural network in the process of network deepening. The distribution of the input value of the deep neural network before nonlinear transformation gradually shifts or changes with the increase in the network depth or during the training process, generally approaching the upper or lower limits of the value interval of the nonlinear function. This leads to the gradient of a shallow neural network disappearing in the case of back propagation, and problems such as training difficulties and slow convergence. Differently, BN forcibly changes the distribution of any input value of each layer of the neural network into a standard normal distribution with a mean value of 0 and a variance of 1 through certain standardized means. In this case, the activated input value falls in the area where the nonlinear function is more sensitive to the input value, and thus, a relatively large gradient can be obtained to avoid the gradient disappearing and realize acceleration of the training. In a YOLOv4-tiny network, BN layers are set behind almost every convolution layer, which is beneficial to model training, but on the other hand, it increases the amount of model computation. Therefore, we propose an optimization method of integrating the BN layer and convolution layer to reduce the amount of computation. The integration process of the BN layer and convolution layer is as follows:

For the convolution layer, the process of convolution is to use the convolution kernel as a sliding window to perform sliding window computation on the corresponding input feature map. Thus, the output formula of this process is:

(1) $$y_{k}^{j}=\sum_{i=1}^{C}w_{k,j}^{j}*x_{i}^{j}+b$$

where $y_{k}^{j}$ is the kth output feature map of the jth layer, $w_{k,j}^{j}$ is the weight of the ith convolution kernel of the kth group of convolution kernels in the jth layer, $x_{i}^{j}$ is the parameter of the feature map of the ith input channel in the jth feature layer, *C* is the number of input channels, and *b* is the bias.

The mean value and variance formulae of the feature map are:

(2) $$\mu =\frac{1}{m}\sum_{i=1}^{m}y_{i}^{j}$$

(3) $$\delta^{2}=\frac{1}{m}\sum_{i=1}^{m}{\left(y_{i}^{j}-\mu \right)}^{2}$$

where $y_{k}^{j}$ is the kth output feature map of the jth layer, m is the number of all elements of the feature map, and μ and δ are, respectively, the mean value and the variance of $y_{i}^{j}$.

Through the mean value and the variance, the normalization formula of the output feature map $y_{k}^{j}$ is obtained as:

(4) $$\hat{y_{i}^{j}}=\frac{y_{i}^{j}-\mu }{\sqrt{\delta^{2}+\varepsilon }}$$

where ε is a minimal value added to prevent the denominator from being zero.

By introducing the zoom variable γ and the translation variable β, which are obtained through model training, we can obtain the processed convolution computation formula:

(5) $$y_{k}^{j}=\gamma *\hat{y_{i}^{j}}+\beta$$

The final convolution computation formula can be obtained by synthesizing the above formulae:

(6) $$y_{k}^{j}=\sum_{i=1}^{C}x_{i}^{j}\times \frac{\gamma \times w_{k,i}^{j}}{\sqrt{\delta^{2}+\varepsilon }}+\frac{\gamma \times (b-\mu )}{\sqrt{\delta^{2}+\varepsilon }}+\beta$$

The above process is the integration of the BN layer and convolution layer.

### 3.2. Sixteen-Bit Fixed-Point Quantization

In hardware design, fixed-point data is common rather than the full precision floating-point data, and the resource consumption of fixed-point data computing units is less than that of floating-point data computing units. There are many advantages in the use of fixed-point quantization of the deep neural network model. First, under the same constraints of hardware resources, the use of low bit fixed-point data for computing can achieve a high degree of computation parallelism to realize fast computing. Second, use of the low bit fixed-point data for computation uses less on-chip storage units. Third, the reduction in the storage resource consumption also greatly reduces the amount of data accessed by external DRAMs and their data bandwidth; so, the energy consumption of the entire hardware system is lowered. Although fixed-point quantization reduces the detection accuracy of the model to a certain extent, as the deep neural network has strong robustness regarding the output accuracy, the decline in the model detection accuracy is still within a reasonable range if the original full-precision floating-point data is replaced with the 16-bit fixed-point data.

This paper performs the 16-bit fixed-point quantization on the weights, biases, and parameters in the input and output feature maps of the algorithm model. The highest bit of the fixed-point quantized data is the sign bit. If the bits of the fractional part are *f* bits, the integer part occupies (15-*f*) bits, and the quantized 16-bit fixed-point computation formula is:

(7) $$D_{fixed16}=\sum_{i=0}^{15}B_{i}\times 2^{-f}\times 2^{i},B_{i}\in \left\{0,1\right\}$$

where $B_{i}$ is the ith bit of the 16-bit fixed-point data.

### 3.3. Pipeline Operation Optimization

In FPGA, there is a certain delay between the input and output. If the logic between the input and output is complex, the delay is relatively long. This requires the time interval between data writing and data reading to be long enough to avoid data reading errors. The existence of this delay seriously affects the speed of FPGA data processing. The idea of a pipeline is to divide a complex logical task into successively different parts. Assuming that a task requires a total of three clock cycles to produce stable results, then the startup interval between each task is composed of three clock cycles. However, the pipeline operation converts this task into three steps, and each step requires a clock cycle to produce results. The output results of the first step are first cached, then used as the input of the second step, and so on. In this way, the first step can proceed to the next task without waiting for the completion of the next two steps. Hence, the system becomes faster with a stronger overall processing capacity.

**Figure 3:**
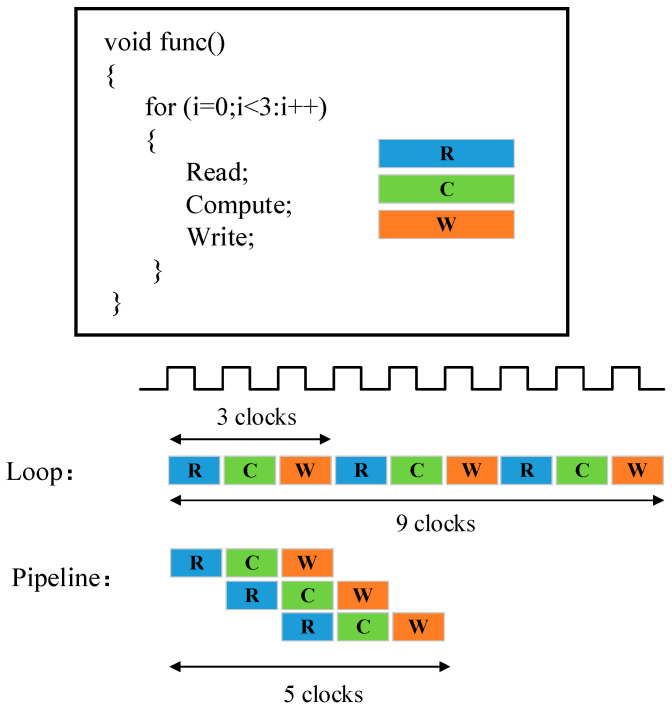
Pipeline instruction optimization structure.

Take the pipeline instruction optimization structure shown in Figure 3 as an example. When the pipeline operation is not used for optimization, a task needs to perform three loops and each loop contains three steps. Thus, each loop will take three clock cycles in total, and the task needs to take nine clock cycles to be completed. However, if pipeline instructions are used, only five clock cycles are needed to complete a task.

### 3.4. Ping-Pong Operation

“Ping-pong operation” is to continuously send the buffered data stream to the data processing unit through the input selection unit and the output selection unit. Therefore, it is very suitable for pipelining data processing. The ping-pong operation can reduce the delay caused by the data input and enable the low-speed module to process high-speed data streams.

**Figure 4:**
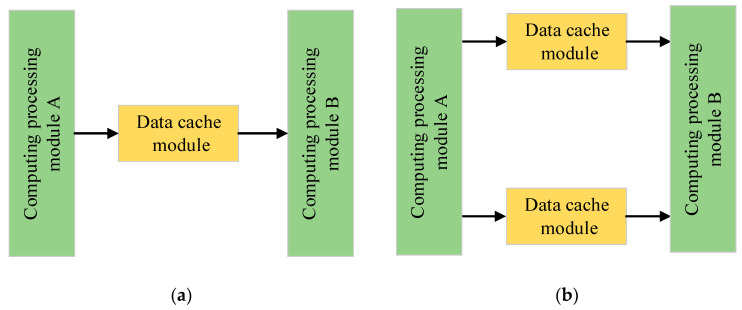
Data processing (**a**) without ping-pong operation and (**b**) with ping-pong operation.

Two types of data processing are shown in Figure 4. When there is no ping-pong operation in data processing, as shown in Figure 4a, it is assumed that two processing modules perform data reading and writing on the data cache module at the same clock frequency. If there is only one data cache module, as the computation processing module cannot simultaneously perform data reading and writing, one computation processing module is always idle. When the ping-pong operation is taken in data processing, as shown in Figure 4b, two data cache modules are deployed between two computation processing modules: module A first writes data into one of the data cache modules, and then module B reads the data from this data cache module and module A simultaneously writes data into the other data cache module. In this way, the two computation processing modules can work together to read and write data alternately.

### 3.5. Parallelism of Input and Output Channels

In convolutional neural networks, the input feature graph of each convolution layer usually contains multiple channels so that each set of involved convolution kernels has the same number of channels as the input. Moreover, the data operation between different channels is independent of each other. Therefore, the computation of the input feature graphs of different channels and that of the corresponding convolution kernels can be designed in parallel.

**Figure 5:**
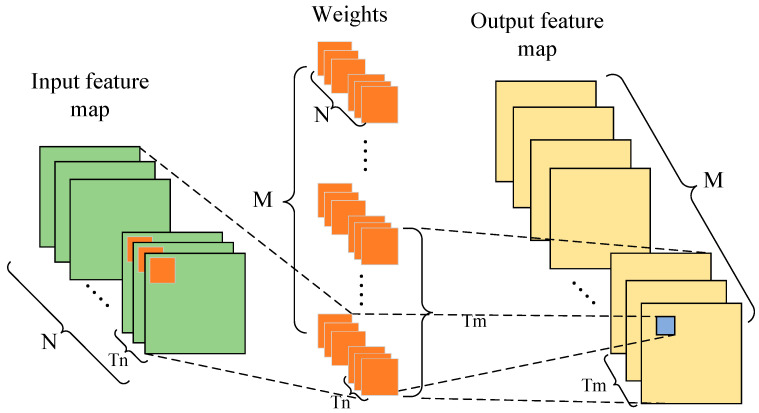
Parallel computation of input and output channels.

A parallel design consumes a large amount of FPGA resources. If the FPGA resources are sufficient, all input channels and output channels can be simultaneously calculated in parallel so that the convolution computation only consumes one convolution kernel and one feature graph to compute the convolution of the whole layer. However, FPGA resources are often limited. To shorten the computing time, it is necessary to make full use of the FPGA resources to allow more input and output channels to be computed in parallel. Figure 5 illustrates the parallel computation of the input and output channels, in which there are N input channels and M output channels, and Tn input channels and Tm output channels are computed in parallel. The process of a parallel computation is as follows:

Operation 1: Tn input channels in a group of N convolution kernels are computed in the form of a sliding window in parallel with Tn input channels in N input feature maps. All results of the computation form an output feature map.

Operation 2: Tm groups of convolution kernels perform Operation 1 at the same time so that Tm output feature graphs can be obtained.

When designing a convolution IP kernel with parallel computation of Tn input channels and Tm output channels, it needs to be multiplexed (MN)/(TnTm) times to compute a complete one-layer convolution. Therefore, the multiplexing times of the IP kernel should be reduced to shorten the computing time, that is, to increase the number of input and output channels for parallel computation as much as possible.

## 4. FPGA Implementation of the YOLOv4-Tiny Algorithm

### 4.1. IP Kernel Design of the Algorithm Acceleration Module

The computation in the YOLOv4-tiny algorithm is mainly composed of convolution and pooling computation that exceeds 90% of the computation amount of the total network, in which convolution computation accounts for the main part. Therefore, in the FPGA implementation of the algorithm, the key lies in the design of the IP kernels for the computation of convolution and pooling. This section introduces the design of convolution and pooling IP kernels using codes and structure diagrams. During the design of the convolution IP kernel, I/O channel parallelism, pipeline, and ping-pong operation are used for optimization. Compared with convolution computing, pooling computing is relatively simple. Therefore, to reduce the FPGA resource consumption, only the pipeline optimization method is used for the design of the pooling IP kernel.

#### 4.1.1. Design of Convolution IP kernel

**Figure 6:**
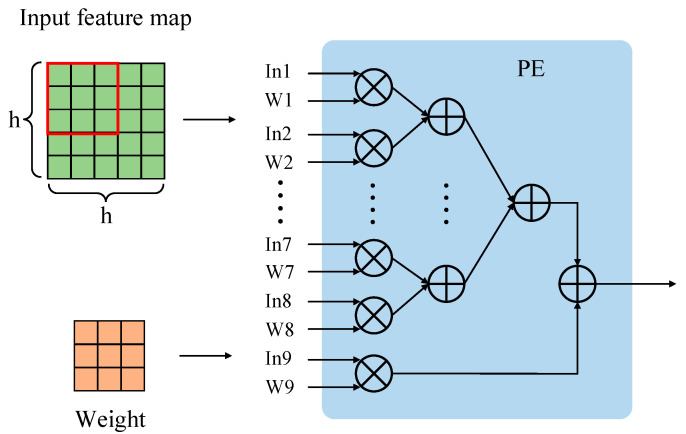
Design of a 3 × 3 convolution operator.

The key to designing the convolution IP kernel is to design a convolution operator. The YOLOv4-tiny algorithm contains 3 × 3 convolutions and 1 × 1 convolutions. Taking the design of the 3 × 3 convolution operator as an example, as shown in Figure 6, the input image is a h × h matrix, and the convolution weight is a 3 × 3 matrix. The weight matrix is convolved with the data matrix at the corresponding position of the input image in the form of a sliding window. Computation of the 3 × 3 convolution is completed by a dot product processing elements PE. Codes are used to describe the implementation of the convolution operator in detail.

**Figure 7:**
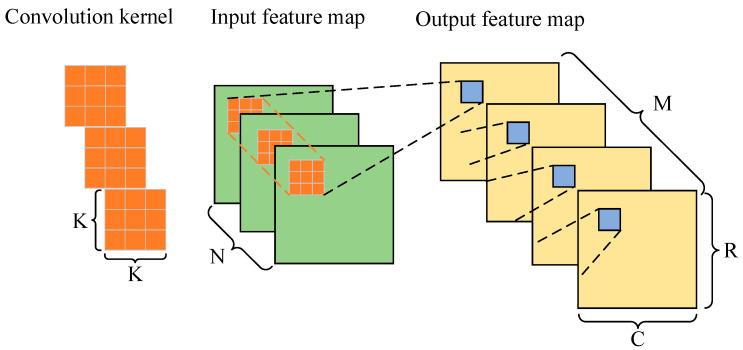
Convolution layer computation.

Generally, the input and output of a convolution layer are composed of multiple two-dimensional feature maps. Figure 7 shows the computation process of a convolution layer with N input channels and M output channels. The size of each output feature map is R × C. The weight parameter is composed of multiple convolution kernels, each with a size of K and number of N × M.

The output of the computation of a convolution layer can be expressed as:

(8) $$fm\_out\_buff[m][r][c]=\sum_{n=0}^{N-1}\sum_{kr=0}^{K-1}\sum_{kc=0}^{K-1}wt\_buff[m][n][kr][kc]\;\times fm\_in\_buff[n][S\times r+kr][S\times c+kc]$$

where fm_out_buff represents the elements in the *r*th row and the *c*th column of the *m*th output feature map, wt_buff refers to the elements in the *kr*th row and the *kc*th column of the convolution kernel that corresponds to the output feature map m and the input feature map *n*, fm_in_buff represents the elements in the S×r+krth row and the S×c+kcth column of the input feature map *n*, and *S* stands for the step size of the convolution.

To implement the above formula with code, we need six layers of embedded for loop, that is the processing elements PE. Here, pseudo codes as Algorithm 1 are adopted for a clear description.
**Algorithm 1. Computation of Convolution Layer**Input: fm_in_buff[n][S*r+kr][ S*c+kc] //Input feature map array   wt_buff[m][n][kr][kc] //Weight parameter array     K, R, C, Tn, Tm //Size of convolution kernel; row and column of output feature             map; unfolded input and output channel numbers  Output: fm_out_buff[m][r][c] //Output feature map array  Algorithm:  #pragma HLS ARRAY_PARTITION variable=wt_buff complete dim=1  #pragma HLS ARRAY_PARTITION variable=wt_buff complete dim=2  #pragma HLS ARRAY_PARTITION variable=fm_out_buff complete dim=1  #pragma HLS ARRAY_PARTITION variable=fm_in_buff complete dim=1   for(kr=0; kr<K; kr++) //Periodically traverse the rows of convolution kernel   for(kc=0; kc<K; kc++) //Periodically traverse the columns of convolution kernel   for(r=0; r<R; r++) //Periodically traverse the rows of output feature map    for(c=0; c<C; c++) //Periodically traverse the columns of output feature map  #pragma HLS PIPELINE II=1   for(mm=0; m<Tm; m++) //Periodically traverse the rows of input feature map  #pragma HLS UNROLL    for(nn=0; n<Tn; n++) //Periodically traverse the columns of input feature map  #pragma HLS UNROLL     fm_out_buff[m][r][c]+=fm_in_buff[n][r*S+kr][c*S+kc]*wt_buff[m][n][kr][kc];

Two optimization methods, pipeline and parallel computation of the input and output channels, are added to the above codes. Pipeline optimization is carried out with the PIPELINE instruction. PIPELINE means reducing the startup interval of a function or loop by allowing concurrent execution of operations. The default startup interval is 1, which means that the system processes a new input every clock cycle. After using the PIPELINE instruction, the computation rate is greatly increased. The parallel computation of the input and output channels requires the use of the UNROLL command to unroll the input feature map channel and output feature map channel in parallel. UNROLL means unrolling the loop to create multiple independent operations. The use of this command in a design creates multiple copies of the loop, which allows some or all loop iterations to occur in parallel. In the above codes, Tn channels in the total N input channels and Tm channels in the total M output channels are unrolled. Tn and Tm can be flexibly configured according to the FPGA hardware resources and network scale. In our design, the maximum values of Tn and Tm can be taken as 4 and 32. Before using the UNROLL command, the input and output feature map arrays need to be split. The command used is ARRAY_PARTITION, which means dividing an array into smaller arrays or individual elements. It is necessary to unroll the input feature map array fm_in_buff and the output feature map array fm_out_buff in the first dimension and unroll the weight parameter array wt_buff in the first and second dimensions.

**Figure 8:**
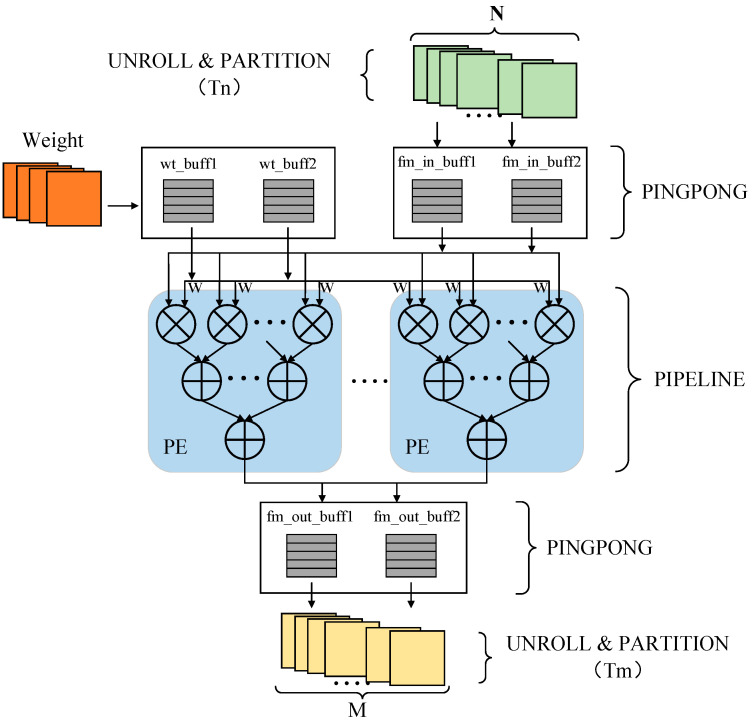
Convolution layer computation structure.

A complete convolution layer computation structure is shown in Figure 8. In the convolution IP kernel design in this paper, parallel and cyclic unrolling is performed on Tn input channels and Tm output channels, and pipeline optimization is taken for the intermediate computing processing unit PE. Moreover, the convolution IP kernel is also optimized from the aspect of the access and store efficiency. Two input feature map data buffers, In_Buffer0 and In_Buffer1, are set between the input feature map and the processing unit PE; two weight parameter input buffers, Wt_Buffer0 and Wt_Buffer1, are set between the weight data and the PE; two output feature map data buffers, Out_Buffer0 and Out_Buffer1, are set between the PE and the output feature map. The ping-pong operation enables each module to work at the same time and perform read and write operations alternately, which greatly improves the processing speed of the system.

#### 4.1.2. Design of Pooling IP kernel

Pooling computation is a very important step in the convolution neural network, and its main function is to reduce dimensionality, remove redundant information, and compress feature maps. Compared with the convolution layer, the pooling layer has no weight parameter and a smaller computation amount. Thus, the design of the pooling IP kernel is simpler than that of the convolution IP kernel. General pooling computation includes average pooling and maximum pooling. The latter is used in the YOLOv4-tiny algorithm. Similar to convolution, pooling is also used in the form of a sliding window to obtain the maximum value from the corresponding positions in the feature maps, and all the maximum values form a new output feature map. In the design of the pooling IP kernel in this paper, the size of the pooling kernel is 2 × 2 and the step size is 2, which can reduce the size of the input feature map to half of the original.

**Figure 9:**
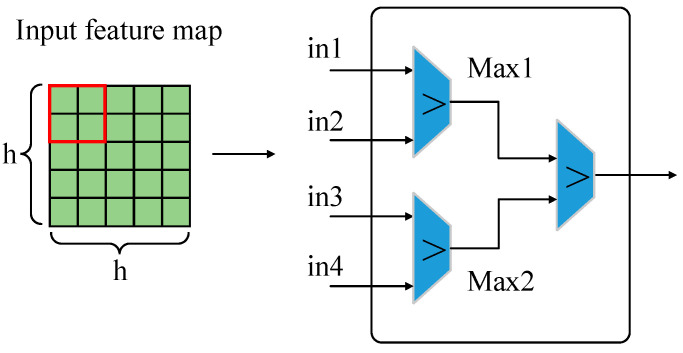
Pooling computation structure.

Figure 9 shows the structure of the pooling computation. The 2 × 2 pooling kernel expressed by a red frame slides on the feature map and takes the four data of the corresponding windows as four inputs for pairwise comparison. In this way, two maximum values, Max1 and Max2, are obtained. Then, the final maximum value can be acquired by comparing Max1 and Max2. In the design of the pooling IP kernel, the pseudo codes adopted are shown as Algorithm 2. Because the pooling computation is relatively simple, only the pipeline optimization is adopted in this design.
**Algorithm 2. Pooling Layer Computation**Input: fm_in_buff[Tn][Tr*2][Tc*2] //Input feature map array  Output: fm_out_buff[Tn][Tr][Tc] //Output feature map array  Algorithm:   for(unsigned short n=0;n<Tn;n++)//Periodically traverse channels of the input feature map   for(unsigned short i=0;i<Tr;i++)// Periodically traverse rows of the input feature map   for(unsigned short j=0;j<Tc;j++)// Periodically traverse columns of the input feature map  #pragma HLS PIPELINE      tmp1=fm_in_buff[n][i*2][j*2];      tmp2=fm_in_buff[n][i*2][j*2+1];      tmp3=fm_in_buff[n][i*2+1][j*2];      tmp4=fm_in_buff[n][i*2+1][j*2+1];      max1=(tmp1>tmp2)?tmp1:tmp2;      max2=(tmp3>tmp4)?tmp3:tmp4;      max=(max1>max2)?max1:max2;      fm_out_buff[n][i][j]=max;

### 4.2. FPGA Hardware System Implementation of the YOLOv4-Tiny Algorithm

**Figure 10:**
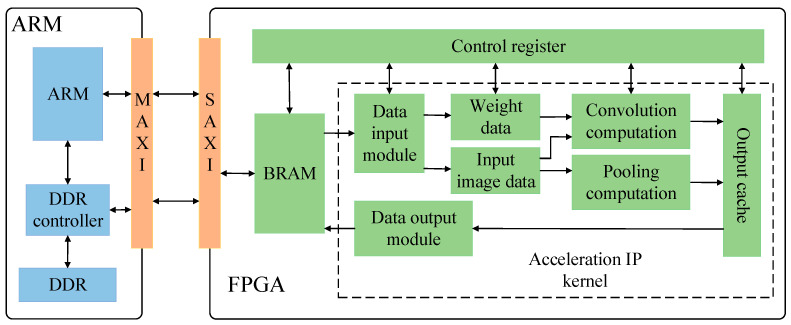
Overall system structure of FPGA implementation.

The system hardware platform is ZYNQ-7020, which adopts the ARM + FPGA heterogeneous computing framework. The overall system structure for the FPGA implementation of the proposed algorithm in this paper is shown in Figure 10, among which the FPGA platform mainly includes convolution, pooling acceleration circuits, data input and output circuits for auxiliary computing, and on-chip cache to complete the parallel computing of the convolution layer and pooling layer; the ARM terminal mainly carries out the work with less computation, such as the initialization of each module, image preprocessing, upper sampling layer, YOLO detection post-processing, and completes the forward reasoning of the entire algorithm network by reusing convolution and pooling IP kernels; BRAM is an FPGA on-chip storage device, which is responsible for storing input and output data; the DDR controller is responsible for controlling the data interaction between the external memory DDR and the AXI bus interface, and storing all interaction data and the final computation results of the algorithm network. Because the weight of the trained algorithm model and the to-be-detected input coal gangue image occupy a large space, it is necessary to use SD cards as the initial storage device, and then read through the DDR later.

The convolution computing unit mainly completes the computation of the convolution and leaky ReLU activation function in the algorithm while the pooling computing unit mainly completes the maximum pooling operation. BRAM interacts with the off-chip DDR through the AXI bus, reads the input data on the off-chip storage required for computation, saves the results to the output cache, and then writes them back to the off-chip DDR through the data output module and the AXI bus. The pooling computing unit and the convolutional computing unit utilize the input and weight buffers through time division multiplexing, thus greatly saving on chip storage resources. The operation scheduling of all modules in the FPGA part is configured by the control register.

## 5. Experiments and Discussion

### 5.1. Experimental Environment

Table 1 shows the experimental environment of the proposed algorithm on the computer platform and FPGA platform.

### 5.2. Computer Platform Experiment and Discussion

#### 5.2.1. Coal Gangue Data Set

**Figure 11:**
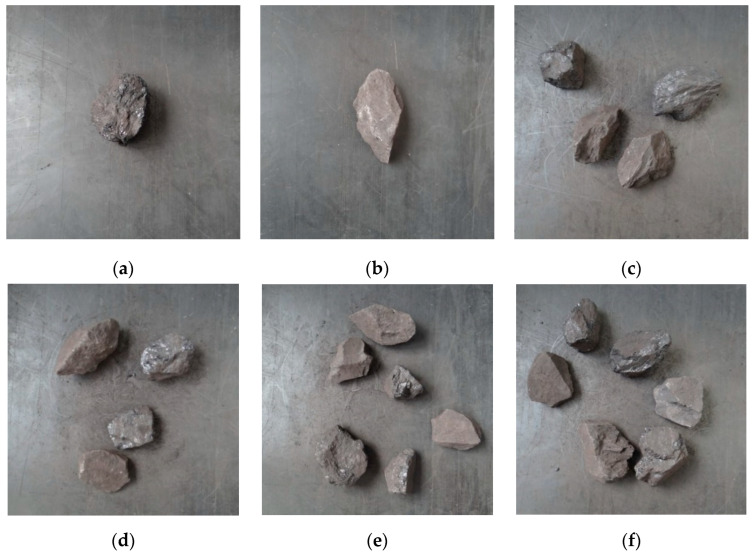
Some images of the coal gauge samples: (**a**) coal; (**b**) gangue; (**c**–**f**) several coal and gangue samples.

There is no open coal gangue data set due to little existing research on deep learning recognition of coal gangue images. Several coal and gangue blocks were collected from coal preparation plants and pictures of them on a background of a mining conveyor belt obtained using traditional cameras. In total, 3852 coal gangue images were obtained and some sample images are shown in Figure 11. In the deep learning training, the detection effect after training usually favors the category with a large number of samples. Therefore, to ensure the balance of the samples, we selected basically the same quantities of coal and gangue for our experiments. To facilitate the training of the network model, the sample images were uniformly cut into 416 × 416 squares.

#### 5.2.2. Algorithm Model Training and Results

In the model training, the training set and verification sets were divided by 8 to 2. The training set included 3082 images and the verification set included 770 images. The training adopted the method of cosine annealing, with a maximum learning rate of 0.02 and a minimum learning rate of 0.0002. The training epoch was set to 100.

**Figure 12:**
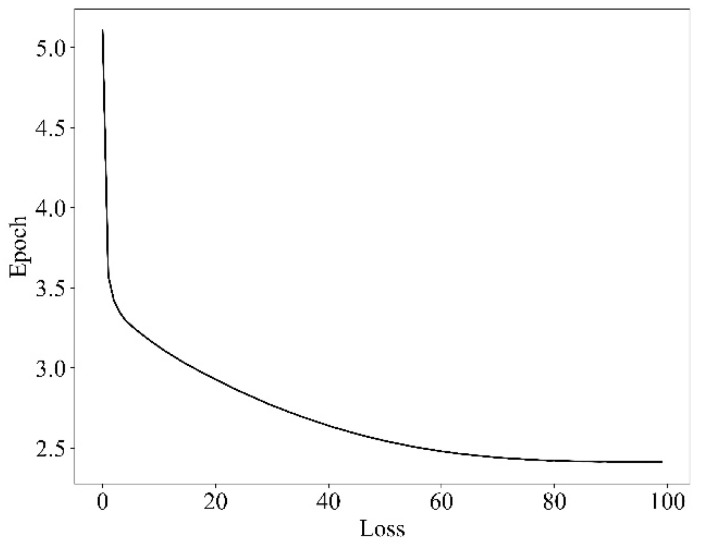
Training epoch–loss diagram.

Figure 12 shows the training epoch–loss curve, from which it can be known that the loss drops sharply in the first 10 epochs, slows down afterwards, and tends to be smooth after 80 epochs. Finally, the loss is about 2.4, indicating that the Loss convergence of the proposed algorithm is fast with a good training effect.

After the training, the weight parameters of the trained algorithm model were saved to the SD card for further FPGA platform experiments.

**Figure 13:**
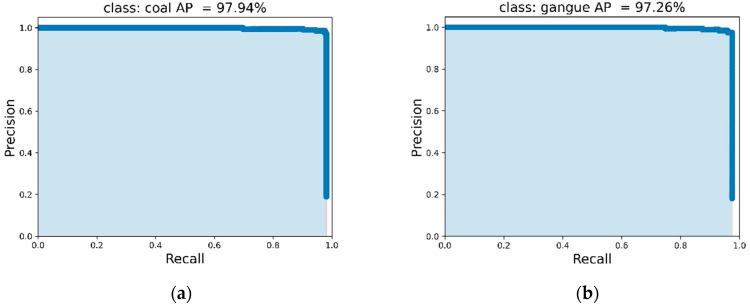
P–R curve of coal and gauge: (**a**) P–R curve of coal; (**b**) P–R curve of gauge.

In this paper, the precision rate precision (P), recall rate recall (R), average precision (AP), and average precision mean (mAP) are used as the recognition accuracy indicators of the algorithm. The (P–R) curve of the YOLOv4-tiny algorithm in this paper under the coal gangue data set is shown in Figure 13, including (a) the (P–R) curve of coal and (b) the P–R curve of gangue. The area between the bottom of the curve and the top of the X-axis is the average recognition precision of the target category. It can be seen from Figure 13 that the average precision of coal and gangue detection is 97.94% and 97.26%, respectively, and an average accuracy mean (mAP) of coal and gangue detection of 97.60% is calculated. This indicates high recognition precision.

### 5.3. FPGA Platform Experiment and Discussion

#### 5.3.1. FPGA Resource Utilization

**Figure 14:**
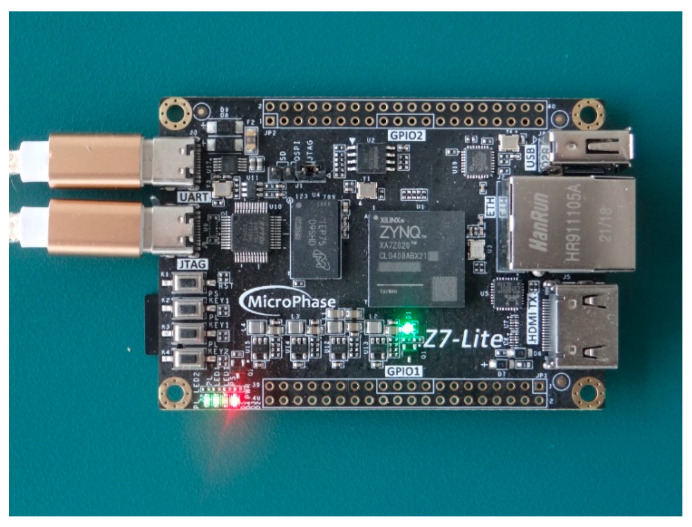
ZYNQ-7020 experimental platform.

The FPGA experimental platform in this paper is ZYNQ-7020, as shown in Figure 14. The platform resources include 53,200 LUTs, 17,400 LUTRAMs, 106,400 FFs, 140 BRAMs, 220 digital signal processors, and 32 global clocks BUFG.

Table 2 shows the utilization the of FPGA resources in this paper. The higher the resource utilization rate, the better the performance of FPGA, and the better the design. The utilization rates of LUT and BRAM are high as they are storage resources while those of LUTRAM, FF, and BUFG, lookup table storage units, triggers, and dedicated clocks, respectively, are relatively low. DSP is the main computing resource to complete the addition and multiplication operations, of which the utilization rate is up to 98%. This indicates that this paper makes full use of FPGA resources and improves the parallelism of the computation as much as possible.

#### 5.3.2. Power Consumption and Performance Analysis

The ZYNQ processor reads the algorithm model weight parameter file in the SD card and the coal gangue image to be detected to complete image recognition. A large number of coal gangue images are tested, and the mAP of coal gangue and the average calculation time of each image are calculated.

For embedded systems, power consumption is an important indicator for ensuring stable and reliable operation of the system. The overall power consumption of the FPGA hardware system in this experiment was 2.86 W.

To verify the performance of the algorithm in this paper on the FPGA platform, experiments were also carried out on the CPU and GPU platforms for comparison. The CPU model is Intel core i5-10200H, and the GPU model is GeForce RTX2060. The performance comparison results of the algorithm on different platforms obtained through the experiments are shown in Table 3.

It takes 0.376 s to recognize a coal gangue image on the FPGA platform, which is between those on CPU and GPU. Since the data precision of both CPU and GPU is 32-bit, their average precision means the coal and gangue image recognition is the same. Due to the 16-bit fixed-point quantization, the average precision mean of the FPGA platform decreases slightly by 1.25%, which is within a reasonable range. The computing performance of the hardware platform can be measured by the GOPS (giga operation per second) indicator, which indicates that one billion times of operation can be performed per second. Although the GOPS of FPGA is lower than those of CPU and GPU, FPGA still achieves the highest energy efficiency ratio due to its low power consumption, which is 10.42 and 3.47 times that of CPU and GPU, respectively.

At present, there are many studies on the implementation of YOLO series algorithms on the FPGA platform. The results of this paper were compared with those of several literatures, as shown in Table 4. The higher the utilization rate of DSP resources, the better the FPGA performance. The utilization rate of DSP resources in this paper is the highest, reaching 98.18%. The utilization rate of DSP resources of Wei [24] is the lowest, less than half. Compared with Yu [25], this paper has a shorter computing time (latency), lower power consumption, and higher energy efficiency ratio. The authors of Li [26] also carried out FPGA implementation of the YOLOv4 tiny algorithm, but it lacks the analysis of GOPS and has high latency.

**Figure 15:**
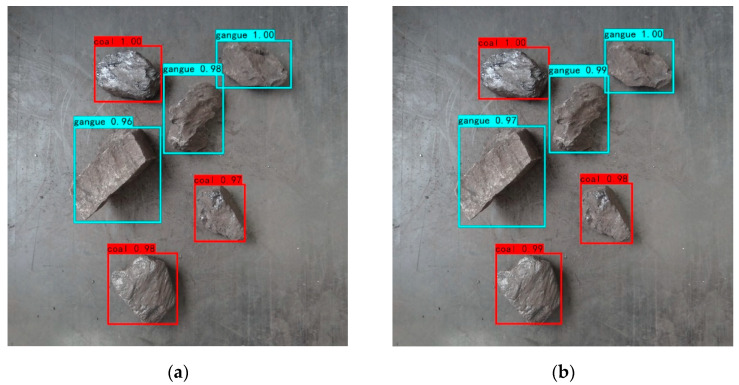
Comparison of the recognition results of coal gangue images on different platforms: (**a**) FPGA platform; (**b**) computer platform.

The average precision of coal and gangue identification on the computer platforms CPU and GPU is the same. To compare the coal gangue image recognition results of the FPGA platform and computer platform, an image of coal gangue blocks, in which coal and gangue, respectively, account for half, is recognized, and the comparison results are shown in Figure 15. The upper left corner of the recognition box in the image shows the type of target identified and the confidence level. The higher the confidence level, the higher the target recognition precision. It can be seen from the figure that the algorithm proposed in this paper correctly identifies all coal gangue targets on both the FPGA platform and the computer platform. Compared with the computer platform, the confidence level of coal gangue identification on the FPGA platform is slightly lower, but all confidence levels are above 0.95, indicating that the FPGA platform can still meet the requirements of high-precision identification under the condition of low power consumption.

## 6. Conclusions

This paper proposes a YOLOv4-tiny-based coal gangue recognition method and used it on the low power consumption hardware platform ZYNQ-7020 to realize fast and accurate recognition of coal gangue images.

(1) In this paper, the integration of the BN layer and convolution layer, and 16-bit fixed-point quantization were performed to initially reduce the computational load of the YOLOv4-tiny model.
(2) In the design of convolution and pooling IP kernels, pipeline and ping-pong operations were adopted to improve the computing speed of the system. In addition, this paper adopted parallel computation of the input and output channels to make full use of the FPGA resources, which accelerated the computation speed.
(3) In the computer platform experiments, the mean average precision (mAP) of coal and gangue was 97.60%. Due to fixed-point quantization, the mAP value on the FPGA platform was 1.25% lower than that on the computer platform, and the recognition time of each image on the FPGA platform was 0.376 s, between that of CPU and GPU. However, the FPGA power consumption was only 2.86 W, much lower than that of CPU and GPU. Although GOPS of FPGA was lower than that of CPU and GPU, FPGA still showed the highest energy efficiency ratio due to its low power consumption, which was 10.42 and 3.47 times higher than that of CPU and GPU, respectively.

The FPGA chip used in this paper was ZYNQ-7020, which has a cheap price, low power consumption, and limited resources. If the identification of coal gangue on site is carried out and the subsequent sorting work is completed, the identification speed needs to be further improved. Later, we can consider the use of FPGA chips with more resources to further improve the parallelism of computing and the recognition speed. The work of this paper has a certain significance to research on the identification and separation equipment of coal gangue on site.

## Figures and Tables

**Table 1:** Environmental environment.

Environment	Description
Windows10	Operating system
Intel core i5-10200H	Processor CPU
NVIDIA GeForce RTX2060(6G)	Video card GPU
DDR4 16G	Memory
Python 3.7	Python version
Tensorflow 2.4.0	Deep learning frame
ZYNQ-7020	FPGA platform

**Table 2:** Utilization of FPGA resources.

Resource	Utilization	Available	Utilization Rate %
LUT	41,953	53,200	78.86
LUTRAM	7414	17,400	42.61
FF	47,652	106,400	44.79
BRAM	96	140	68.57
DSP	216	220	98.18
BUFG	1	32	3.13

**Table 3:** Performance comparison results of the algorithm on the FPGA, CPU, and GPU platforms.

Experimental Platform	CPU	GPU	FPGA
Data precision	32-bit	32-bit	16-bit
Computing time per image/s	0.495	0.065	0.376
Average precision mean (mAP)	97.60%	97.60%	96.35%
GOPS	14.04	106.92	9.24
Power/w	45	115	2.86
Energy efficiency ratio (GOPs/w)	0.31	0.93	3.23

**Table 4:** Performance comparison between this paper and FPGA implementation of other YOLO series algorithms.

	YOLO [24]	YOLOv3-Tiny [25]	YOLOv4-Tiny [26]	This Paper
platform	ZYNQ Board	Zedboard	ZYNQ-7020	ZYNQ-7020
Latency/s	-	0.532	18.025	0.376
DSP/total	409/900	160/220	149/220	216/220
GOPS	-	10.45	-	9.24
Power/w	7.518	3.36	2.384	2.86
GOPs/w	-	3.11	-	3.23

## Data Availability

The data that support the findings of this study are available on request from corresponding author.
